# Knowledge mapping and emerging trends in pediatric hemiplegia research: a bibliometric study spanning 1982–2025

**DOI:** 10.3389/fneur.2025.1590937

**Published:** 2025-07-17

**Authors:** Xiaoli Li, Ya Guo, Lingchuan Niu

**Affiliations:** ^1^Department of Rehabilitation, Children’s Hospital of Chongqing Medical University, National Clinical Research Center for Child Health and Disorders, Ministry of Education Key Laboratory of Child Development and Disorders, Chongqing Key Laboratory of Child Neurodevelopment and Cognitive Disorders, Chongqing, China; ^2^Department of Rehabilitation, The Second Affiliated Hospital, Chongqing Medical University, Chongqing, China

**Keywords:** pediatric hemiplegia, bibliometric analysis, research trends, citation analysis, international collaboration

## Abstract

**Background:**

Pediatric hemiplegia is a significant neurological condition that impacts motor function and quality of life. This bibliometric analysis aimed to evaluate research trends, collaboration patterns, and emerging topics in pediatric hemiplegia research.

**Methods:**

Publications were retrieved from the Web of Science Core Collection database spanning from 1982 to 2025. The analysis was conducted using VOSviewer, CiteSpace, and R-bibliometrix to examine contributions of countries, institutions, authors, journals, and keywords.

**Results:**

A total of 1,840 publications were analyzed, showing consistent growth with an annual growth rate of 8.69%. The United States emerged as the leading contributor with 393 publications, followed by Italy (137) and the United Kingdom (124), with strong international collaboration networks evident among 64 countries. The University of London demonstrated the highest institutional productivity with 142 publications, while Royal Children’s Hospital showed the strongest collaborative connections. *Developmental Medicine* and *Child Neurology* ranked as the most productive journal with 195 publications and highest citation impact. Boyd RN and Gordon AM were identified as the most influential authors based on h-index metrics and collaboration strength. Keyword analysis revealed five distinct research clusters, with “alternating hemiplegia,” “mutations,” “classification,” and “risk factors” emerging as current research hotspots since 2017.

**Conclusion:**

This bibliometric analysis provides a comprehensive overview of research progress and identifies key hotspots in pediatric hemiplegia research, revealing the field’s evolution from basic clinical descriptions to advanced genetic and classification studies. These findings offer valuable insights for researchers and clinicians to understand current research priorities and guide future investigations in pediatric hemiplegia management and treatment strategies.

## Introduction

Pediatric hemiplegia, also known as hemiparetic cerebral palsy, is a unilateral motor impairment that typically results from damage to the developing brain, affecting one side of the body (1). This condition occurs in approximately 0.3–0.6 per 1,000 live births and represents one of the most common subtypes of cerebral palsy in children (2). The impact of pediatric hemiplegia extends beyond motor dysfunction, often encompassing cognitive, sensory, and social developmental challenges that significantly affect both the affected children and their families (3). The etiology of pediatric hemiplegia is diverse, including perinatal stroke, congenital brain malformations, infections, and genetic factors (4).

Over the past several decades, research in pediatric hemiplegia has evolved significantly, from pathogenesis to innovative therapeutic approaches. The introduction of constraint-induced movement therapy (CIMT) and bimanual training has marked important milestones in rehabilitation strategies (5). Additionally, advanced neuroimaging techniques have enhanced our understanding of brain plasticity and recovery mechanisms in young patients (6). Recent years have witnessed an exponential growth in research output related to pediatric hemiplegia, accompanied by increasing international collaboration and interdisciplinary approaches (7–9). The emergence of new therapeutic modalities, including robotics, virtual reality, and non-invasive brain stimulation, has opened new avenues for treatment (10). Furthermore, the focus has expanded to include quality of life measures and long-term functional outcomes, reflecting a more holistic approach to patient care (11).

Bibliometric analysis, as a quantitative approach to analyzing academic literature, can reveal research patterns, identify influential contributors, and highlight emerging trends in a field (12). Such analysis is particularly valuable in medical research, as it can guide future research directions, inform funding allocation, and facilitate productive collaborations (13). Several bibliometric studies have been conducted in related fields. Wang et al. (14) analyzed global research trends in spastic cerebral palsy, providing accurate and expedited insights into critical information and potentially new directions in the study of spastic CP. Wu et al. (15) conducted a bibliometric analysis of brain imaging in children with cerebral palsy, revealing information regarding mechanism, prognosis, and therapeutic efficacy in the field of CP research. However, no comprehensive bibliometric analysis specifically focusing on pediatric hemiplegia research has been conducted to date. The present study aims to provide a systematic bibliometric analysis of pediatric hemiplegia research from 1982 to 2025, focusing on publication trends, international collaboration networks, institutional contributions, and research hotspots.

## Materials and methods

### Data sources and search strategy

Publications were retrieved from the Web of Science Core Collection database (WOSCC), which provides extensive coverage across various disciplines, covering the timespan from January 1, 1982, to June 6, 2025. The search strategy employed the following terms: TS = (“Hemiplegia*” OR “Hemiplegic Paralysis”) AND TS = (“infant*” or “child*” or “childhood” or “pediatric” or “toddler*” or “bab*” or “trottie” or “kid*” or “neonate*” or “newborn*” or “adolescent*” or “teenager” or “juvenile*” or “teen*”). This comprehensive search strategy was designed to capture all relevant publications related to pediatric hemiplegia. English was the publication language for this study, with only articles being considered from the range of document types. Information including the number of publications, citations, titles, author data, institutions, countries/regions, keywords, and journals was compiled for further bibliometric analysis.

### Data analysis

For bibliometric and knowledge mapping analysis, VOSviewer (version 1.6.20), CiteSpace (version 6.3.R1), and R-bibliometrix (version 4.3.3) were utilized. VOSviewer is a bibliometric analysis software that can extract key information from numerous publications, which is often used to build collaboration, co-citation, and co-occurrence networks (16). In this study, the software mainly completes the following analysis: country and institution analysis, journal and co-cited journal analysis, author and co-cited author analysis, and keyword co-occurrence analysis.

Due to differences in data scale across categories, uniform thresholds were not adopted to maintain visual clarity and interpretability. To ensure clarity, different inclusion thresholds were applied based on the data volume of each category: countries ≥ 1 documents, institutions ≥ 7, authors ≥ 4, journals ≥ 3, and keywords ≥ 15 occurrences.

CiteSpace (version 6.3.R1) is another software developed for bibliometric analysis and visualization (17). CiteSpace was applied to map the keyword analysis and to analyze references with Citation Bursts. For keyword analysis, the node type was set to keywords with selection criteria of top 5 items per slice and pruning using pathfinder network scaling. The R package “bibliometrix”^1^ was applied for thematic evolution analysis and to construct global distribution networks of publications on pediatric hemiplegia research (18). The quartile and impact factor of journals were obtained from Journal Citation Reports 2023. Additionally, Microsoft Office Excel 2022 was used to conduct quantitative analysis of publications. The h-index, m-index, and g-index were used to assess the academic impact of both individuals and journals. The h-index is a key tool for evaluating researchers’ scholarly contributions and offers insight into their future scientific achievements (19, 20). The m-index, which adjusts the h-index based on the number of years since an author’s first publication, was used to identify early-career researchers with high potential. The g-index measures the cumulative impact of a researcher’s work by ranking publications by citations in decreasing order (21). The Impact Factor (IF) serves as a journal-level metric measuring the yearly average number of citations to recent articles published in that journal.

## Results

### Overall characteristics

**Figure 1:**
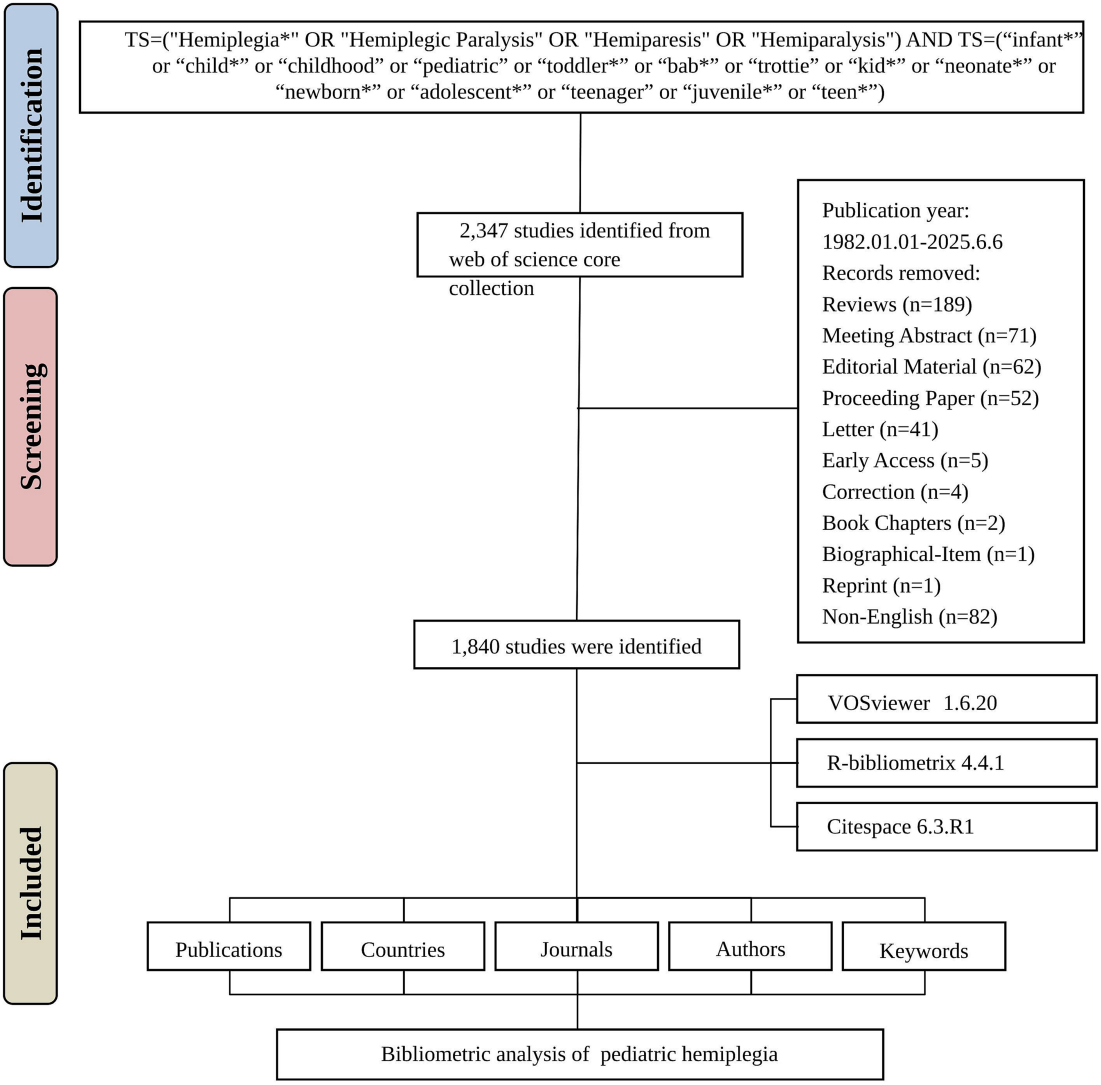
Flowchart of the literature screening process.

**Figure 2:**
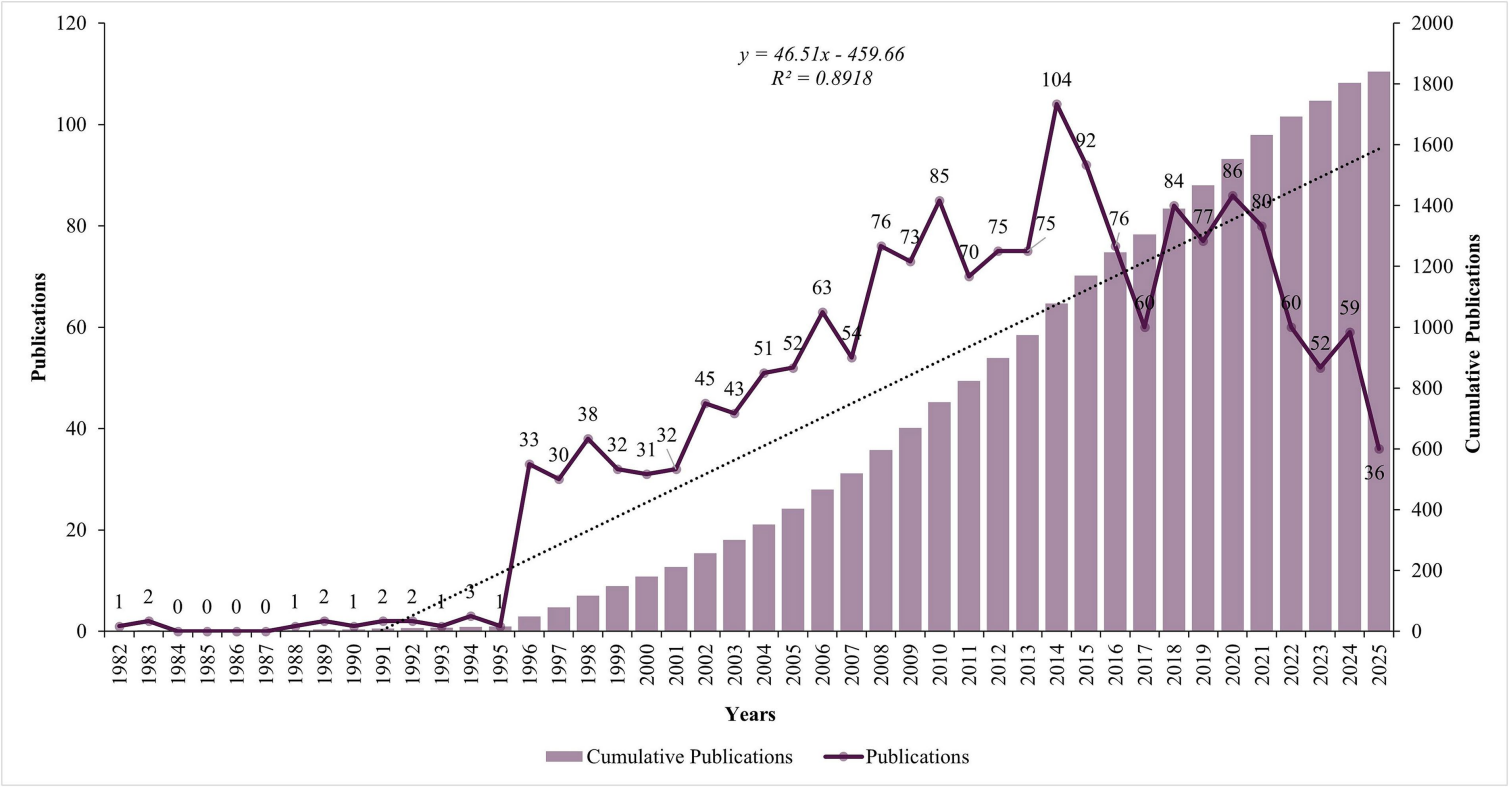
Annual number of publications.

A total of 2,347 studies were initially identified from the WOSCC between 1982 and 2025. After removing duplicate records and applying inclusion/exclusion criteria, 1840 studies were included in the final analysis (Figure 1). The field experienced minimal research activity during its early years (1982–1995), with sporadic publications and long periods of inactivity. A significant transformation began in 1996, marking the emergence of sustained research interest with 33 publications, followed by steady growth through the late 1990s and early 2000s. The research field demonstrated remarkable expansion until reaching a peak of 104 publications in 2014. Notable fluctuations occurred in recent years, with publication numbers declining from the 2014 peak to 36 publications in 2025 (Figure 2).

### Analysis of countries

**Figure 3:**
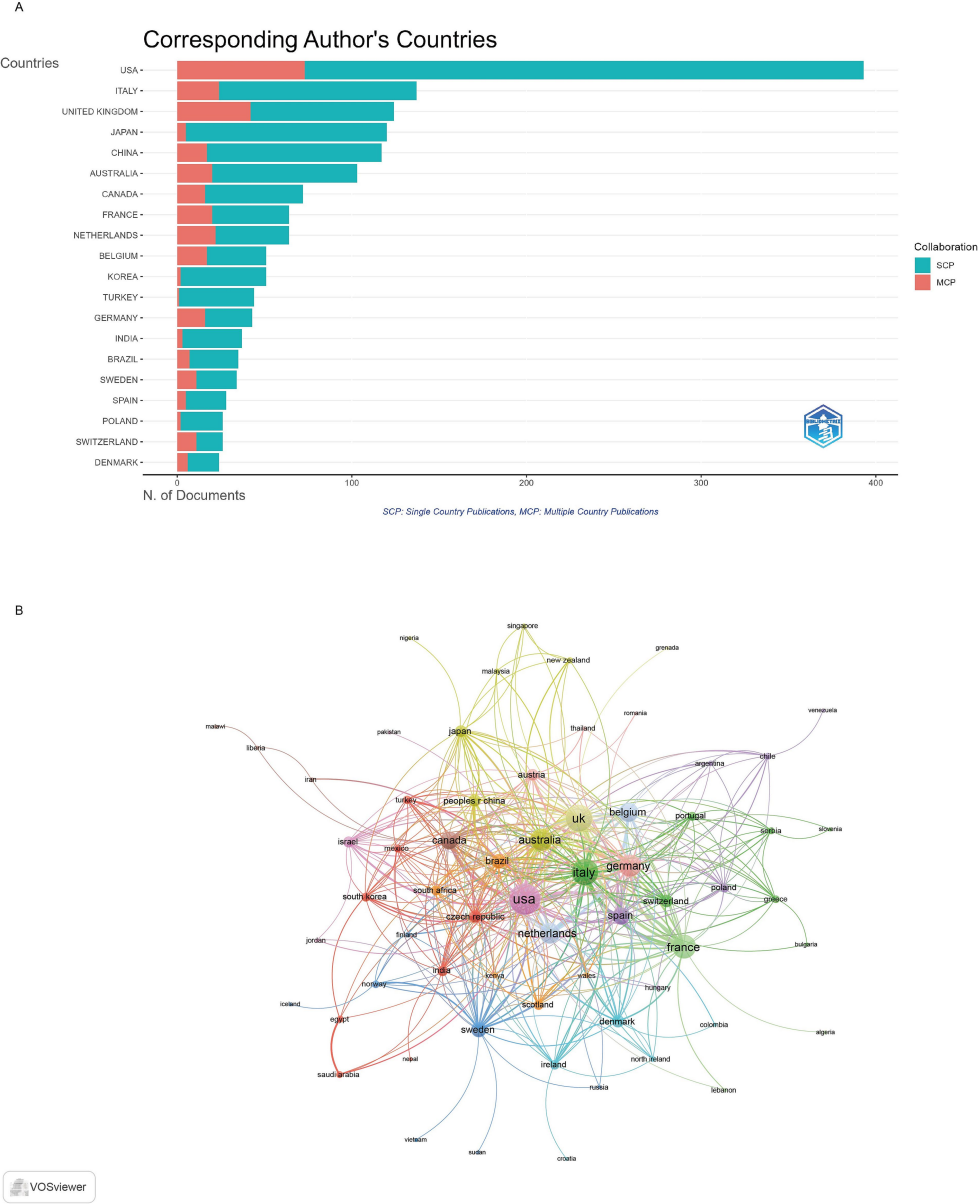
Analysis of countries. **(A)** Distribution of corresponding author’s publications by country. **(B)** Visualization map depicting the collaboration among different countries.

The United States emerged as the undisputed leader in research productivity with 393 publications (TP = 1,548, TC = 15,419), representing 21.4% of all corresponding author publications in the field. Italy secured the second position with 137 publications (TP = 575, TC = 2,831), accounting for 7.4% of global output, while the United Kingdom ranked third with 124 publications (TP = 438, TC = 6,064), contributing 6.7% of total publications (Figure 3A). The publication and citation profiles of the top 20 countries are presented in Supplementary Table S1. Sweden demonstrates the highest average citation rate of 107.4 citations per publication despite having only 34 publications. The United Kingdom follows with 48.9 average citations per publication. Denmark achieves 42.5 average citations per publication, while France (40.4) and Australia (39.7) also demonstrate above-average citation impacts. The United States, despite its dominant publication volume, achieves 39.2 average citations per publication. The visualization map in Figure 3B depicts the collaboration networks among different countries. Countries with a minimum of 1 articles were included, resulting in 64 countries being mapped. The United States had the highest total link strength, collaborating with 308 other countries, followed by the United Kingdom (237) and Italy (223). The top 10 countries by international collaboration strength were listed in Supplementary Table S2.

### Analysis of institutions

**Figure 4:**
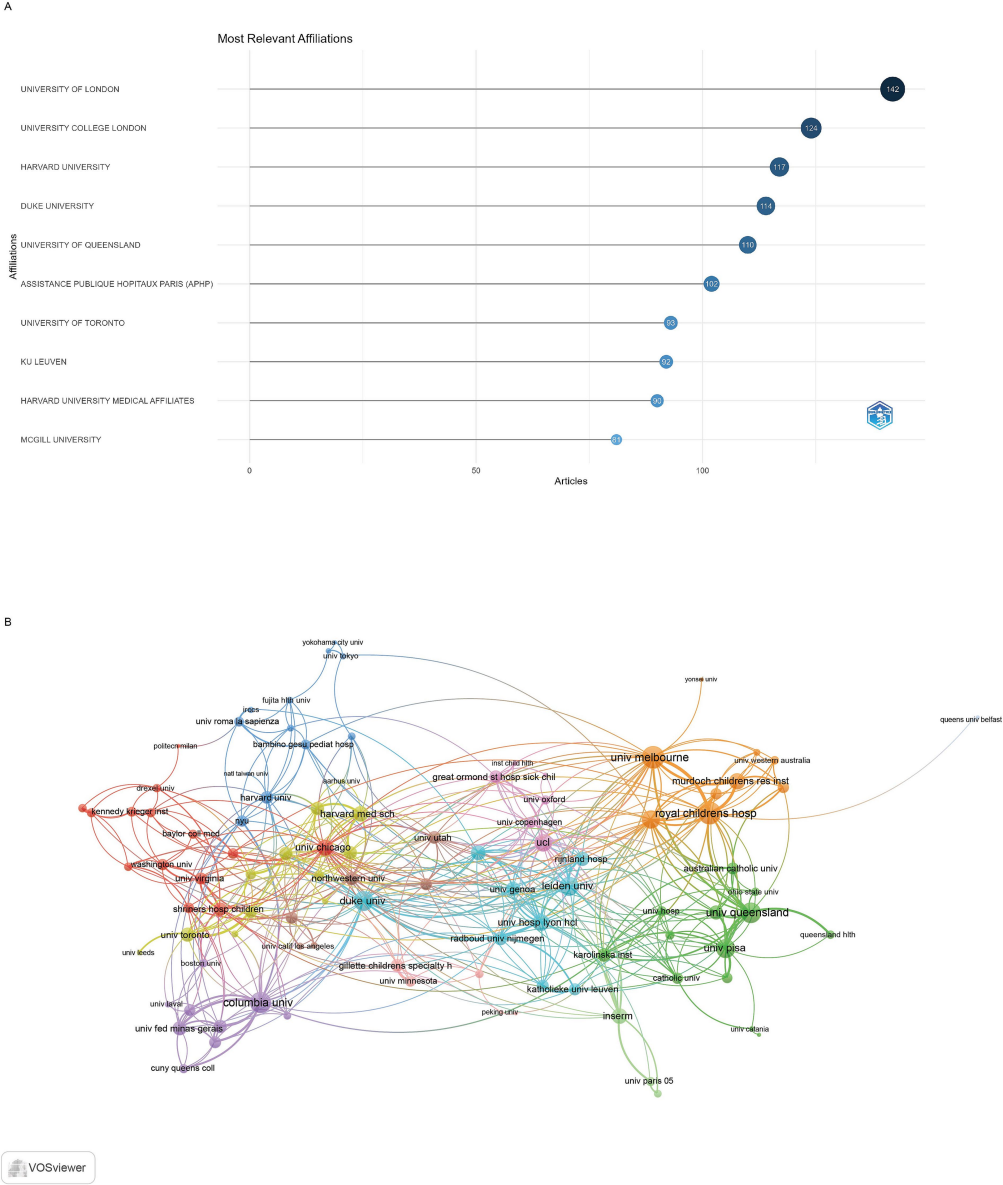
Analysis of institutions. **(A)** Top ten institutions by article count and rank. **(B)** Visualization map depicting the collaboration among different institutions.

The 1840 publications were contributed by authors affiliated with 100 institutions worldwide. University of London was the most productive institutions, with 142 articles, followed by the University College London with 124 articles and Harvard University with 117 articles (Figure 4A). The visualization map depicting the collaboration networks among different institutions reveals that Royal Children’s Hospital and University of Melbourne had the highest number of collaborations with other institutions (64 each), followed by Columbia University (57) (Figure 4B).

### Analysis of journals

The top 20 most productive journals in this field are listed in Supplementary Table S3. *Developmental Medicine* and *Child Neurology* emerges as the premier journal in the field with 195 publications, establishing itself as the primary venue for pediatric hemiplegia research. This journal also demonstrates exceptional citation influence with 5,247 total citations (TC_rank = 1) and maintains the highest h-index of 68, reflecting both productivity and sustained citation impact. *Gait & Posture* ranks second in publication volume with 55 articles and achieves 1,225 total citations (TC_rank = 3), while *Brain & Development* secures third place with 57 publications but achieves 493 total citations (TC_rank = 15). *Brain* leads in impact factor with 10.6 (Q1), followed by *Neurology* with 7.7 (Q1) and *Epilepsia* with 6.6 (Q1), indicating these journals’ positions within the highest-tier publications in neuroscience and neurology.

**Figure 5:**
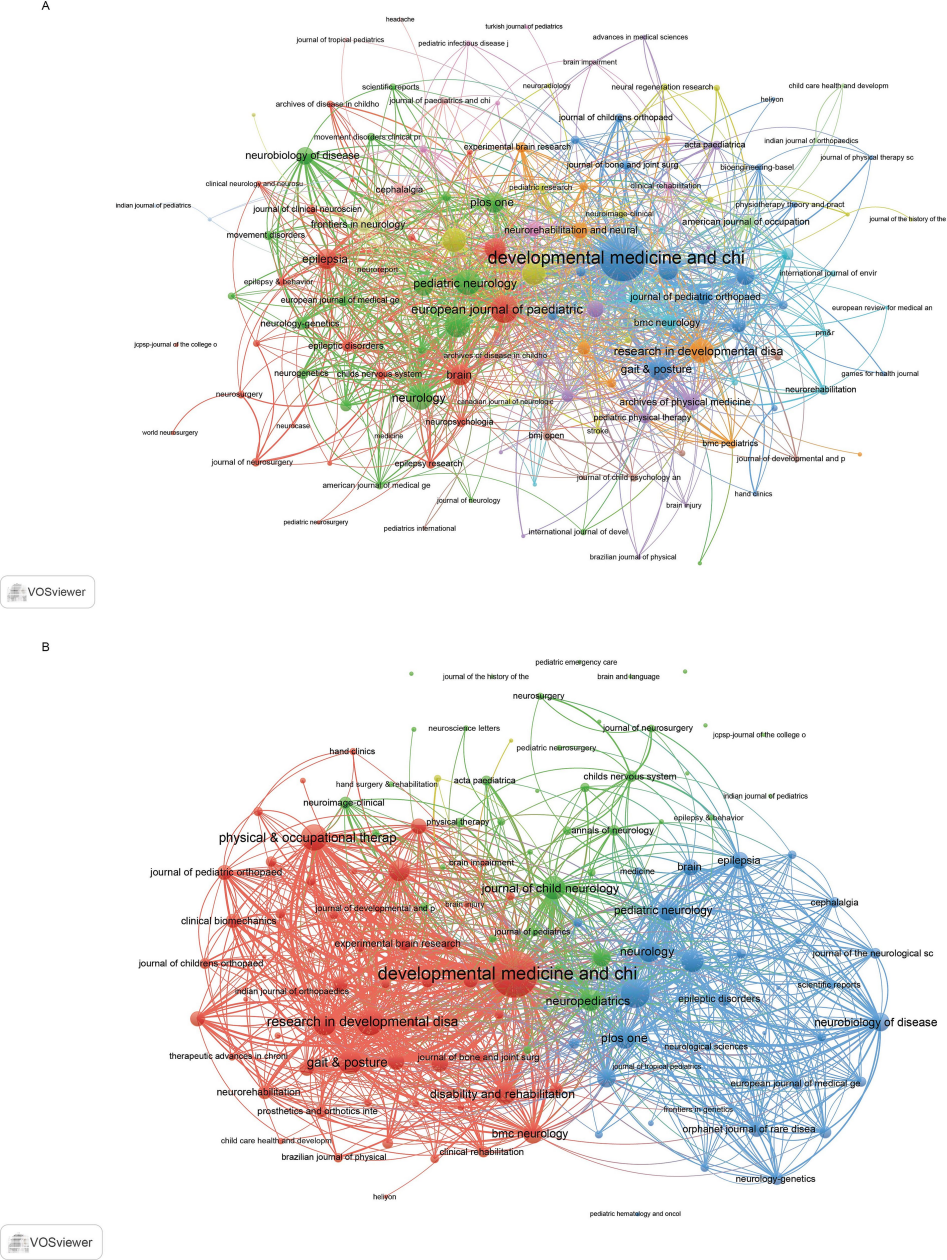
Analysis of journals. **(A)** Co-occurrence network of journals. **(B)** Coupling network of journals.

In the co-occurrence network (Figure 5A), which included 135 journals with at least three occurrences, the three key journals with the highest total link strength were *Developmental Medicine and Child Neurology* (873), *European Journal of Paediatric Neurology* (308), and *Pediatric Neurology* (262). The coupling network (Figure 5B), which also included 138 journals with at least three couples, identified *Developmental Medicine and Child Neurology* (30010), *European Journal of Paediatric Neurology* (11018), and *Research in Developmental Disabilities* (9575) as the key journals with the highest total link strength.

### Analysis of authors

**Figure 6:**
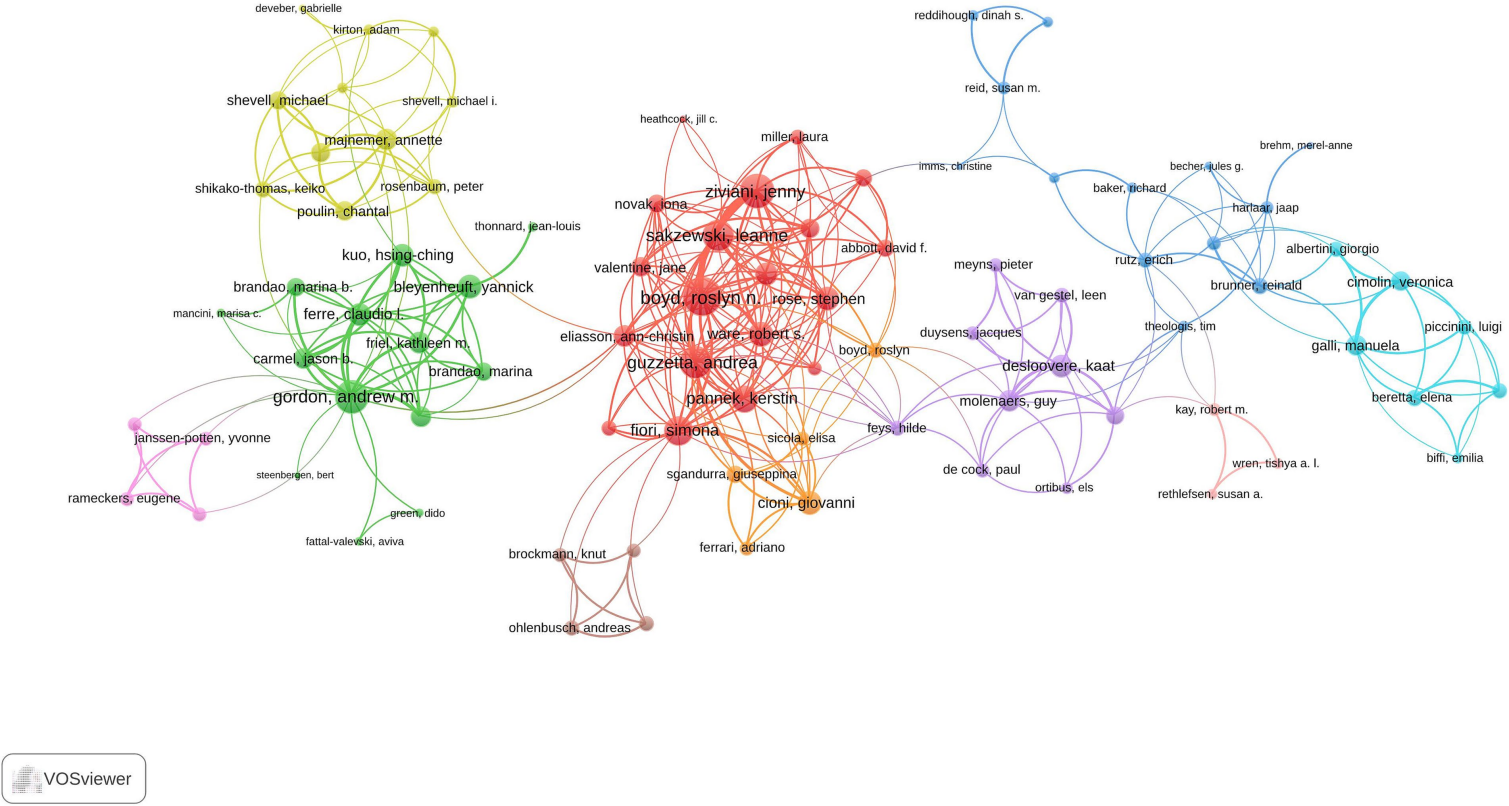
Visualization map depicting the collaboration among different authors.

All publications were authored by 7,857 researchers, with 43 single-authored documents. Supplementary Table S4 presents the publication and citation profiles of the top 20 most impactful authors based on their h-index, which takes into account both the productivity and citation impact of their publications. Boyd RN (TP = 32; TC = 1,744; H-index = 23) emerged as the most influential researcher, followed by Gordon AM (TP = 34; TC = 1,223; H-index = 21) and Cioni G (TP = 23; TC = 1,490; H-index = 19). The collaboration networks among authors are depicted in the visualization map presented in Figure 6. The author collaboration network analysis encompassing 89 authors with a minimum of four articles reveals sophisticated research partnerships and knowledge-sharing patterns within the pediatric hemiplegia research community. Boyd RN (100 total link strength) demonstrates the highest collaboration strength, followed by Mikati MA (93 total link strength) and Ziviani Jenny (82 total link strength).

### Most cited articles

The most cited article in the field of pediatric hemiplegia research was “The Manual Ability Classification System (MACS) for children with cerebral palsy: scale development and evidence of validity and reliability” by Eliasson et al., published in *Developmental Medicine & Child Neurology* (IF = 3.8) in 2006 (22). This article accumulated a total of 1,610 citations. The second most cited article, with 523 citations, was “The Gait Deviation Index: a new comprehensive index of gait pathology” by Schwartz and Rozumalski, published in *Gait & Posture* (IF = 2.2) in 2008 (23). The third most cited article was “International League Against Epilepsy classification and definition of epilepsy syndromes with onset in childhood: Position paper by the ILAE Task Force on Nosology and Definitions” by Specchio et al., published in *Epilepsia* (IF = 6.6) in 2022 (24). This article received a total of 444 citations. A more comprehensive list of the top 20 most cited articles is provided in Supplementary Table S5.

### Analysis of keywords

**Figure 7:**
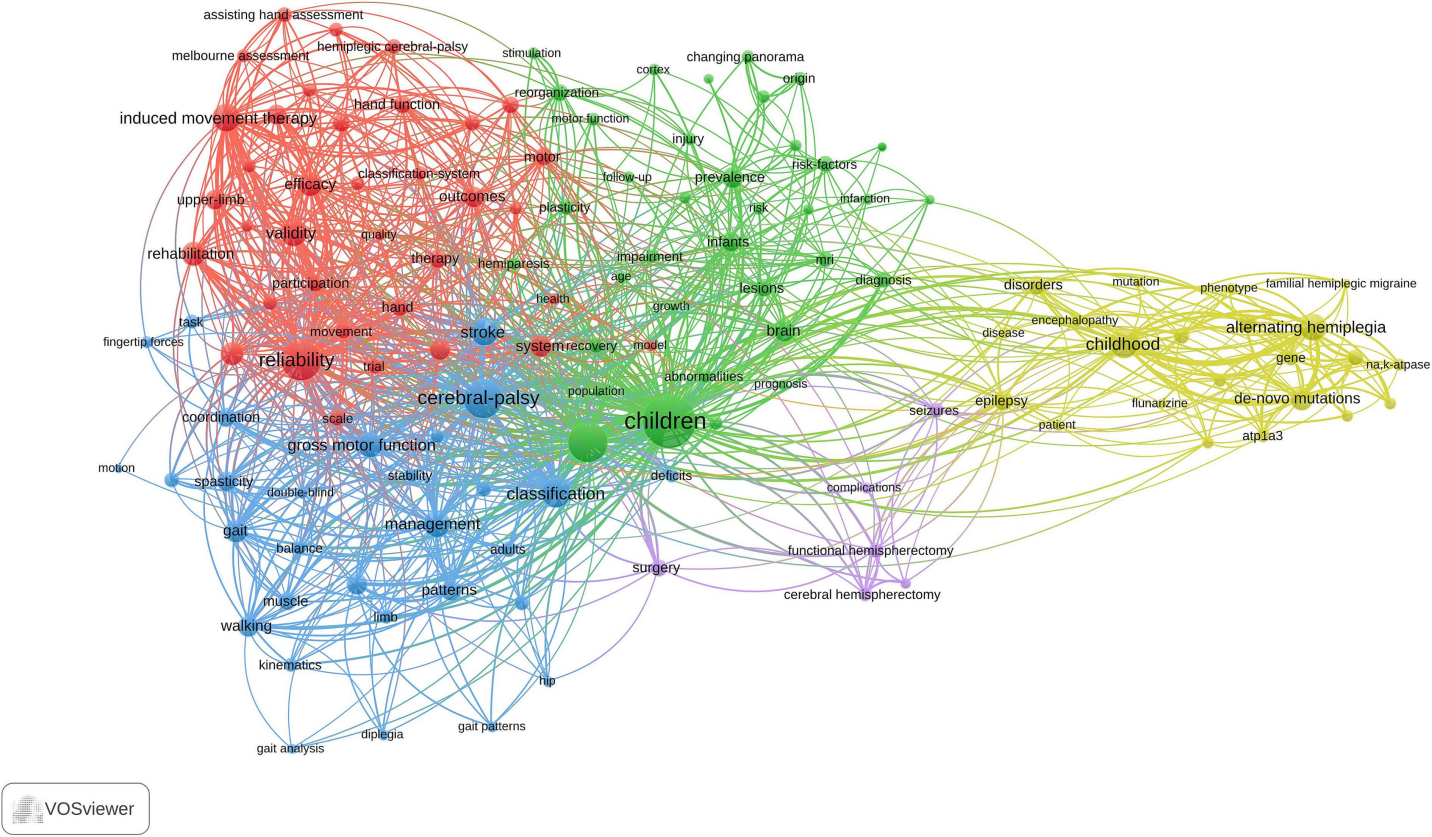
Visual analysis of keyword co-occurrence network analysis.

A total of 130 high-frequency keywords were identified and categorized into five thematic clusters, each reflecting a critical aspect of pediatric hemiplegia research (Figure 7). The red cluster centers on assessment and rehabilitation interventions, highlighting evidence-based therapeutic approaches for motor function improvement. Key terms include “induced movement therapy,” “rehabilitation,” “efficacy,” “validity,” “reliability,” and “randomized controlled trial.” This cluster emphasizes the field’s commitment to developing and validating effective therapeutic interventions through rigorous clinical research methodologies. The green cluster focuses on neurological assessment and motor function evaluation, encompassing comprehensive diagnostic and functional assessment approaches. Keywords in this category include “brain,” “motor function,” “gross motor function,” “gait,” “walking,” “spasticity,” and “lesions.” This cluster reflects the fundamental importance of systematic neurological evaluation in understanding the pathophysiology and functional impact of pediatric hemiplegia. The blue cluster emphasizes clinical classification and management strategies, featuring terms such as “cerebral palsy,” “classification,” “coordination,” “deficits,” “diplegia,” “spastic hemiplegia,” and “management.” This cluster represents the clinical foundation of pediatric hemiplegia research, focusing on diagnostic criteria, classification systems, and comprehensive care approaches that guide treatment decisions and prognosis. The yellow cluster pertains to genetic and molecular mechanisms underlying pediatric hemiplegia, with key terms including “alternating hemiplegia,” “ATP1A3,” “mutations,” “*de novo* mutations,” “gene,” “childhood,” and “mouse model.” This cluster represents the most recent advancement in the field, reflecting the transition from purely clinical approaches to molecular understanding and precision medicine strategies. The purple cluster focuses on surgical interventions and associated complications, featuring terms such as “surgery,” “seizures,” “complications,” “functional hemispherectomy,” “cerebral hemispherectomy,” and “infantile hemiplegia.” This cluster highlights the specialized surgical management options available for severe cases of pediatric hemiplegia, particularly those with medically refractory epilepsy.

**Figure 8:**
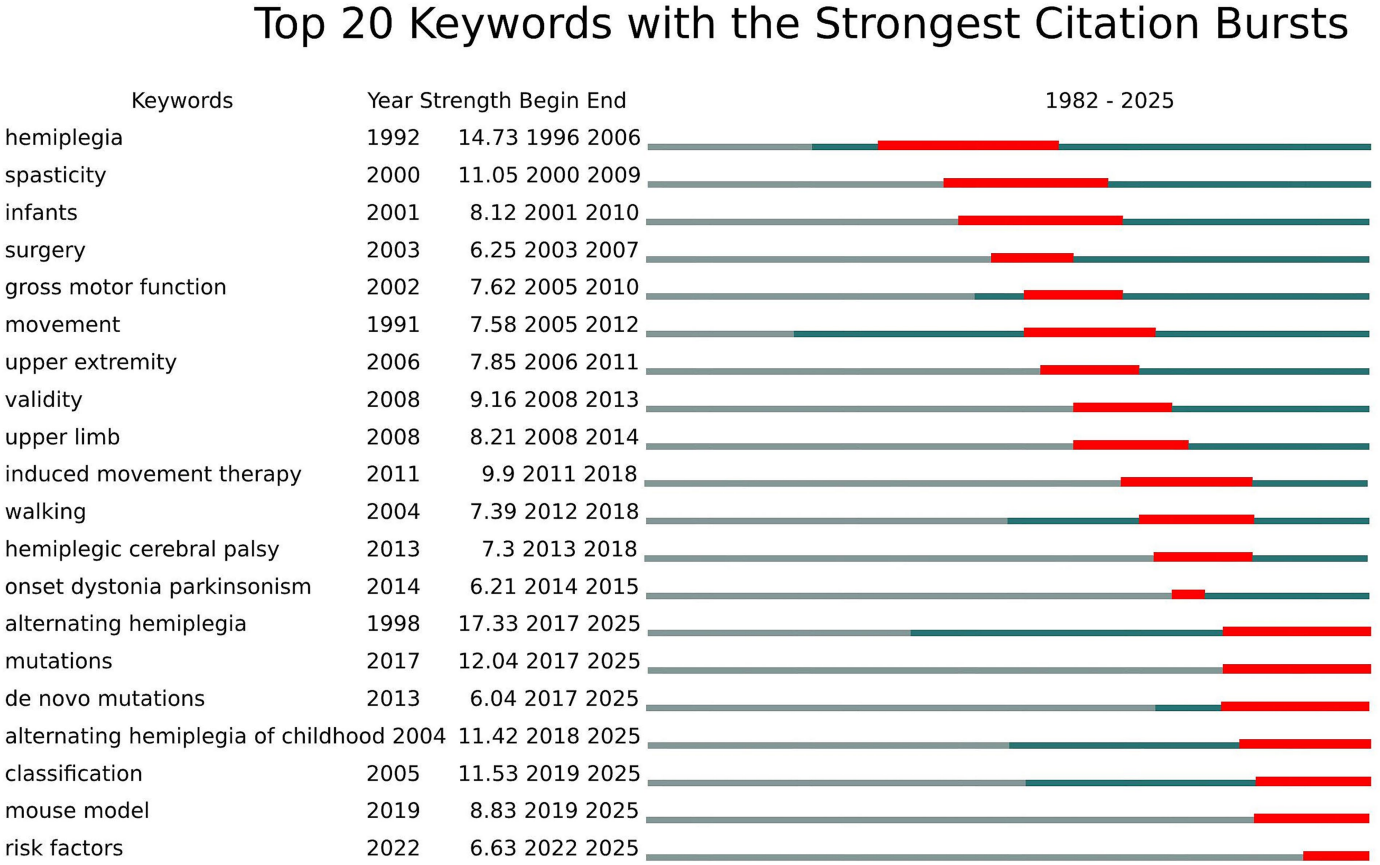
Top 20 Keywords with the strongest citation bursts.

The analysis of the top 20 keywords with the strongest citation bursts from 1992 to 2025 reveals distinct evolutionary phases in pediatric hemiplegia research priorities (Figure 8). The keyword with the highest burst strength was “alternating hemiplegia” (17.33, 2017–2025), followed by “mutations” (12.04, 2017–2025) and “classification” (11.53, 2019–2025). Early bursts primarily focused on basic clinical characterization and traditional therapeutic approaches, as reflected by keywords such as “hemiplegia” (14.73, 1996–2006), “spasticity” (11.05, 2000–2009), and “surgery” (6.25, 2003–2007).

The intermediate period emphasized functional assessment and evidence-based interventions, demonstrated by keywords such as “gross motor function” (7.62, 2005–2010), “induced movement therapy” (9.9, 2011–2018), “validity” (9.16, 2008–2013), and “upper extremity” (7.85, 2006–2011). This period marked the establishment of standardized assessment tools and the development of constraint-induced movement therapy as a leading intervention. In the most recent years, research focus has dramatically shifted toward genetic mechanisms and precision medicine approaches, as demonstrated by emerging keywords such as “alternating hemiplegia” (17.33, 2017–2025), “*de novo* mutations” (6.04, 2017–2025), “alternating hemiplegia of childhood” (11.42, 2018–2025), “mouse model” (8.83, 2019–2025), and “risk factors” (6.63, 2022–2025), all of which continue to show strong bursts through 2025. This transition reflects the field’s evolution from descriptive clinical studies to molecular understanding and translational research approaches.

## Discussion

### General information

This comprehensive bibliometric analysis of 1,840 publications spanning 1982–2025 reveals the dynamic evolution of pediatric hemiplegia research from traditional clinical approaches to precision medicine paradigms. Five distinct research hotspots emerged: assessment and rehabilitation interventions, neurological evaluation and motor function, clinical classification and management, genetic and molecular mechanisms, and surgical interventions. The field demonstrates clear temporal transitions, progressing from basic clinical characterization (1996–2009) through evidence-based intervention development (2010–2018) to the current genetic revolution (2017–2025). The discovery of ATP1A3 mutations in alternating hemiplegia of childhood represents a watershed moment, fundamentally transforming research priorities and opening new avenues for targeted therapies. This evolution reflects broader trends in pediatric neurology toward personalized medicine approaches, with citation burst analysis revealing “alternating hemiplegia,” “mutations,” and “classification” as the strongest emerging research themes continuing through 2025.

The United States’ dominance with 393 publications reflects substantial federal investment through the National Institutes of Health and National Institute of Neurological Disorders and Stroke, which have prioritized pediatric neurological conditions through dedicated funding mechanisms (25). American leadership is further strengthened by major pediatric hospitals and academic medical centers with established cerebral palsy programs and multidisciplinary teams. Italy’s second-place position (137 publications) stems from strong European Union research framework programs and Italy’s historical emphasis on pediatric rehabilitation research, particularly through institutions like Stella Maris Foundation and University of Pisa. The United Kingdom’s contribution (124 publications) reflects the National Health Service’s systematic approach to pediatric neurology care and substantial investment in cerebral palsy research through organizations like Cerebra and Action Medical Research (26). The University of London’s institutional leadership (142 publications) is anchored by Great Ormond Street Hospital’s internationally recognized pediatric neurology program and the Institute of Child Health’s research excellence. Harvard University’s prominence reflects its comprehensive Children’s Hospital network and substantial NIH funding for pediatric neurological disorders research. The Royal Children’s Hospital’s high collaborative strength demonstrates Australia’s strategic focus on pediatric rehabilitation research and strong international partnerships with European and North American institutions.

*Developmental Medicine and Child Neurology*’s position as the leading journal (195 publications) reflects its specific focus on pediatric neurological conditions and rehabilitation, making it the preferred venue for constraint-induced movement therapy research and cerebral palsy classification studies. The journal’s editorial policy favoring evidence-based intervention studies aligns perfectly with the field’s emphasis on therapeutic efficacy research (27). *Gait & Posture*’s prominence indicates the field’s strong biomechanical research component, particularly in analyzing movement patterns and surgical outcomes in hemiplegic children. *Neuropediatrics* serves as a key venue for genetic discoveries, especially ATP1A3-related research, reflecting its editorial preference for molecular neurology investigations (28).

Boyd RN’s leadership in publications and citations reflects her pioneering work in constraint-induced movement therapy for pediatric populations at the University of Queensland, establishing evidence-based protocols that transformed rehabilitation practices globally (29). Gordon AM’s contributions center on bimanual coordination research and the development of Hand-Arm Bimanual Intensive Training, representing crucial advances in upper limb rehabilitation approaches (30). Cioni G’s influence stems from comprehensive clinical phenotyping studies and longitudinal outcome research at University of Pisa, contributing significantly to understanding natural history and prognostic factors in pediatric hemiplegia. The emergence of genetic researchers in recent citation patterns reflects the field’s shift toward molecular investigations, with authors like Mikati MA and Panagiotakaki E leading international consortiums focused on alternating hemiplegia of childhood research and ATP1A3 mutations (31).

### Research hotspots and frontier trends

Based on the keyword clustering analysis, five distinct research clusters were identified in pediatric hemiplegia research, representing current hotspots and emerging trends in the field.

### Cluster 1 (red): assessment and rehabilitation interventions

This cluster focuses on evidence-based assessment tools and therapeutic interventions for pediatric hemiplegia, encompassing key terms such as “induced movement therapy,” “rehabilitation,” “efficacy,” “validity,” “reliability,” and “randomized controlled trial.” Constraint-induced movement therapy (CIMT) has emerged as a prominent intervention, with systematic reviews demonstrating significant improvements in upper limb function in children with hemiplegic cerebral palsy (32). The Manual Ability Classification System (MACS) has become a gold standard for assessing hand function in children with cerebral palsy, providing reliable classification that guides treatment decisions (33). Recent advances include modified CIMT protocols that are more feasible for clinical implementation while maintaining therapeutic efficacy (34). Bimanual training approaches have also gained attention, with studies showing comparable outcomes to CIMT for improving bilateral hand coordination (35). Virtual reality-based interventions represent an emerging frontier, offering engaging and motivating rehabilitation experiences that enhance motor learning through neuroplasticity mechanisms (36). The integration of robotics and technology-assisted therapy continues to evolve, with evidence supporting their use as adjunct treatments to conventional therapy approaches (37).

### Cluster 2 (green): neurological assessment and motor function

This cluster encompasses comprehensive neurological evaluation and motor function assessment, including terms such as “brain,” “motor function,” “gross motor function,” “gait,” “walking,” “spasticity,” and “lesions.” The Gross Motor Function Classification System (GMFCS) serves as a fundamental tool for categorizing functional abilities and predicting long-term outcomes in children with cerebral palsy (38). Gait analysis has become increasingly sophisticated, with three-dimensional gait analysis providing detailed insights into movement patterns and informing surgical decision-making (39). Neuroimaging advances, particularly diffusion tensor imaging, have enhanced understanding of white matter integrity and its relationship to motor outcomes. Spasticity management has evolved beyond traditional approaches, with selective dorsal rhizotomy and intrathecal baclofen showing promising results for appropriate candidates (40). The concept of minimal brain dysfunction has been refined through advanced neuroimaging techniques, revealing subtle structural and functional abnormalities that correlate with clinical presentation (41). Single-event multilevel surgery has gained acceptance as an effective approach for addressing multiple gait abnormalities simultaneously in ambulatory children with cerebral palsy (42).

### Cluster 3 (blue): clinical classification and management

This cluster addresses clinical classification systems and management strategies, featuring terms including “cerebral palsy,” “classification,” “coordination,” “deficits,” “diplegia,” “spastic hemiplegia,” and “management.” The evolution of cerebral palsy classification has moved toward more functional and descriptive systems that better capture the heterogeneity of the condition (43). The International Classification of Functioning, Disability and Health (ICF) framework has been increasingly applied to pediatric hemiplegia, providing a comprehensive approach to understanding disability and participation (44). Evidence-based clinical practice guidelines have been developed to standardize care approaches and improve outcomes. Multidisciplinary team management has become the standard of care, involving neurologists, orthopedic surgeons, physiatrists, therapists, and other specialists working collaboratively (45). Early intervention programs have demonstrated significant benefits in optimizing developmental outcomes when implemented within critical periods of brain plasticity (46). The concept of activity and participation has gained prominence, shifting focus from purely medical models to functional outcomes that matter to children and families (47). Transition planning from pediatric to adult care has emerged as a critical area requiring systematic approaches to ensure continuity of care.

### Cluster 4 (yellow): genetic and molecular mechanisms

This cluster represents the cutting-edge research in genetic and molecular aspects of pediatric hemiplegia, including “alternating hemiplegia,” “ATP1A3,” “mutations,” “*de novo* mutations,” “gene,” “childhood,” and “mouse model.” Alternating hemiplegia of childhood (AHC) has been revolutionized by the discovery of ATP1A3 mutations as the primary cause, occurring in over 80% of cases (48). This breakthrough has enabled precise genetic diagnosis and opened avenues for targeted therapies. *De novo* mutations account for the majority of AHC cases, explaining the typically sporadic occurrence of this condition (49). Mouse models carrying ATP1A3 mutations have provided crucial insights into disease mechanisms and potential therapeutic targets. The phenotypic spectrum associated with ATP1A3 mutations extends beyond classic AHC to include rapid-onset dystonia parkinsonism and other movement disorders (50). Genetic counseling has become an essential component of care, helping families understand inheritance patterns and recurrence risks. Precision medicine approaches are being developed, with potential for sodium-potassium ATPase modulators as targeted therapies (51). The establishment of patient registries and natural history studies has accelerated research progress and clinical trial readiness (52).

### Cluster 5 (purple): surgical interventions and complications

This cluster encompasses surgical management approaches and associated complications, featuring terms such as “surgery,” “seizures,” “complications,” “functional hemispherectomy,” “cerebral hemispherectomy,” and “infantile hemiplegia.” Hemispherectomy procedures have undergone significant refinement, with functional hemispherectomy techniques reducing morbidity while maintaining seizure control efficacy (53). Patient selection criteria have been refined based on long-term outcome studies, emphasizing the importance of early intervention in appropriate candidates. Seizure outcomes following hemispherectomy remain excellent, with seizure freedom rates exceeding 80% in well-selected patients (54). Cognitive and developmental outcomes have been better characterized, showing that early surgery often results in better long-term cognitive function compared to medically refractory epilepsy (55). Minimally invasive techniques, including laser interstitial thermal therapy, represent emerging alternatives for selected cases (56). Complications have become increasingly rare with improved surgical techniques and perioperative management, though long-term monitoring remains essential (57). The concept of brain plasticity and reorganization following hemispherectomy continues to fascinate researchers, with neuroimaging studies revealing remarkable adaptive changes (58). Quality of life measures have become important outcome metrics, demonstrating significant improvements in most patients following successful surgery (59).

Citation burst analysis reveals the evolution of pediatric hemiplegia research priorities across distinct temporal phases, reflecting shifts from basic clinical characterization to advanced genetic understanding and precision medicine approaches.

#### Early clinical foundation (1996–2009)

The initial research phase was dominated by fundamental clinical concepts, with “hemiplegia” showing the strongest burst (strength 14.73, 1996–2006), establishing the field’s foundational knowledge base. During this period, “spasticity” (2000–2009) emerged as a key research focus, reflecting early emphasis on understanding and managing the primary motor manifestations of pediatric hemiplegia. Research concentrated on describing clinical presentations, developing classification systems, and establishing basic therapeutic approaches for managing spasticity in children with hemiplegic conditions (60).

#### Functional assessment and intervention era (2010–2018)

A significant paradigm shift toward systematic functional evaluation and evidence-based interventions characterized this period. Key bursts included “induced movement therapy” (2011–2018), “upper extremity” (2006–2011), “validity” (2008–2013), and “gross motor function” (2005–2010). This era marked the development of constraint-induced movement therapy (CIMT) as a leading intervention for pediatric hemiplegia, with pivotal studies demonstrating its efficacy in improving upper limb function (61). The emphasis on “validity” reflected the field’s commitment to developing reliable assessment tools and standardized outcome measures for clinical trials (62).

#### Genetic revolution (2019–2025)

The most recent phase represents a paradigm shift toward molecular understanding, with “alternating hemiplegia” showing exceptional burst strength (17.33, 2017–2025), followed by “mutations” (2017–2025) and “*de novo* mutations” (2017–2025). The discovery of ATP1A3 mutations in alternating hemiplegia of childhood in 2012 revolutionized the field, leading to genetic-based diagnostic approaches and precision medicine strategies (63). Concurrent emergence of “classification” (2019–2025), “mouse model” (2019–2025), and “risk factors” (2022–2025) indicates the field’s current focus on translational research, genetic counseling, and personalized therapeutic interventions (64).

### Strengths and limitations

This study has several unique advantages. First, we systematically analyzed research spanning nearly six decades, providing a comprehensive overview of the field’s evolution. Second, the use of multiple bibliometric tools (VOSviewer, CiteSpace, and R-bibliometrix) ensured robust analysis of publication patterns and research trends. Third, our analysis of citation patterns and emerging keywords offers valuable insights for identifying promising research directions.

However, this study also has limitations. The data were sourced solely from the WoSCC database, potentially missing relevant publications in other databases like Scopus or PubMed. Although the Web of Science database is widely utilized in bibliometric research for its extensive coverage of high-impact journals and reliable citation data (65), dependence on a single data source may lead to selection bias. By focusing on English-language publications, we may have overlooked contributions in other languages. Additionally, publications from 2025 may be incomplete due to the time of data collection. Due to variations in institutional naming and automatic extraction by different bibliometric tools, standardization of affiliations across analyses could not be fully achieved. In future research, incorporating interviews or qualitative methods to gain deeper insight into this field should be considerate.

## Conclusion

This bibliometric analysis based on cluster analysis identified five research hotspots in pediatric hemiplegia research: assessment and rehabilitation interventions, neurological assessment and motor function, clinical classification and management strategies, genetic and molecular mechanisms, and surgical interventions and complications. There is a clear evolution among the strongest citation bursts, with the period from 1996–2009 primarily concentrated on basic clinical characterization and spasticity management. The intermediate period from 2010–2018 emphasized functional assessment and evidence-based therapeutic interventions, particularly constraint-induced movement therapy. From 2017 onwards, the field has undergone a paradigmatic shift toward genetic mechanisms and precision medicine approaches, with alternating hemiplegia of childhood research leading this transformation.

## Data Availability

The original contributions presented in the study are included in the article/Supplementary material, further inquiries can be directed to the corresponding authors.

## Footnotes

^1^ https://www.bibliometrix.org
