# Supplementary material for: Knowledge mapping and emerging trends in pediatric hemiplegia research: a bibliometric study spanning 1982–2025
Source: Front Neurol. 2025 Jul 17;16:1590937. doi: 10.3389/fneur.2025.1590937 (PMC12310594; doi:10.3389/fneur.2025.1590937)
Supplement: Supplementary file 1 [file Supplementary_file_1.docx]

**Table S1 Publication and Citation Profiles of Leading Countries**

| **Country** | **Articles** | **Freq** | **SCP** | **MCP** | **MCP_Ratio** | **TP** | **TP_rank** | **TC** | **TC_rank** | **Average Citations** |
| --- | --- | --- | --- | --- | --- | --- | --- | --- | --- | --- |
| USA | 393 | 0.214 | 320 | 73 | 0.186 | 1548 | 1 | 15419 | 1 | 39.2 |
| ITALY | 137 | 0.074 | 113 | 24 | 0.175 | 575 | 2 | 2831 | 5 | 20.7 |
| UK | 124 | 0.067 | 82 | 42 | 0.339 | 438 | 5 | 6064 | 2 | 48.9 |
| JAPAN | 120 | 0.065 | 115 | 5 | 0.042 | 438 | 4 | 2249 | 9 | 18.7 |
| CHINA | 117 | 0.064 | 100 | 17 | 0.145 | 369 | 7 | 1388 | 12 | 11.9 |
| AUSTRALIA | 103 | 0.056 | 83 | 20 | 0.194 | 522 | 3 | 4089 | 3 | 39.7 |
| CANADA | 72 | 0.039 | 56 | 16 | 0.222 | 355 | 8 | 2610 | 7 | 36.2 |
| FRANCE | 64 | 0.035 | 44 | 20 | 0.313 | 388 | 6 | 2583 | 8 | 40.4 |
| NETHERLANDS | 64 | 0.035 | 42 | 22 | 0.344 | 276 | 9 | 2640 | 6 | 41.2 |
| BELGIUM | 51 | 0.028 | 34 | 17 | 0.333 | 201 | 10 | 1616 | 10 | 31.7 |
| KOREA | 51 | 0.028 | 49 | 2 | 0.039 | 138 | 12 | 842 | 16 | 16.5 |
| TURKEY | 44 | 0.024 | 43 | 1 | 0.023 | 138 | 13 | 816 | 17 | 18.5 |
| GERMANY | 43 | 0.023 | 27 | 16 | 0.372 | 161 | 11 | 1403 | 11 | 32.6 |
| INDIA | 37 | 0.02 | 34 | 3 | 0.081 | 99 | 17 | 488 | 19 | 13.2 |
| BRAZIL | 35 | 0.019 | 28 | 7 | 0.2 | 111 | 15 | 445 | 20 | 12.7 |
| SWEDEN | 34 | 0.018 | 23 | 11 | 0.324 | 109 | 16 | 3650 | 4 | 107.4 |
| SPAIN | 28 | 0.015 | 23 | 5 | 0.179 | 130 | 14 | 252 | 26 | 9 |
| POLAND | 26 | 0.014 | 24 | 2 | 0.077 | 60 | 22 | 367 | 21 | 14.1 |
| SWITZERLAND | 26 | 0.014 | 15 | 11 | 0.423 | 87 | 20 | 776 | 18 | 29.8 |
| DENMARK | 24 | 0.013 | 18 | 6 | 0.25 | 88 | 18 | 1020 | 13 | 42.5 |

Note(s): Articles: Publications of Corresponding Authors only. Freq: Frequence of Total Publications. MCP_Ratio: Proportion of Multiple Country Publications. TP: Total Publications. TP_rank: Rank of Total Publications. TC: Total Citations. TC_rank: Rank of Total Citations. Average Citations: The average number of citations per publication.

**Table S2.** **Top 10 Countries by International Collaboration Strength.**

| Rank | Country | Documents | Citations | Total link strength |
| --- | --- | --- | --- | --- |
| 1 | USA | 480 | 18080 | 308 |
| 2 | UK | 166 | 8038 | 237 |
| 3 | Italy | 192 | 6330 | 223 |
| 4 | France | 106 | 4495 | 181 |
| 5 | Australia | 146 | 6213 | 159 |
| 6 | Germany | 70 | 2297 | 153 |
| 7 | Netherlands | 99 | 4640 | 134 |
| 8 | Belgium | 77 | 2512 | 117 |
| 9 | Canada | 105 | 5770 | 100 |
| 10 | Spain | 45 | 791 | 94 |

**Table S3 Bibliometric Indicators of High-Impact Journals**

| **Journal** | **H_index** | **G_index** | **M_index** | **IF 2023** | **JCR 2023** | **TP** | **TP_rank** | **TC** | **TC_rank** | **PY_start** |
| --- | --- | --- | --- | --- | --- | --- | --- | --- | --- | --- |
| DEVELOPMENTAL MEDICINE AND CHILD NEUROLOGY | 68 | 107 | 2.061 | 3.8 | Q1 | 195 | 1 | 5247 | 1 | 1993 |
| GAIT & POSTURE | 26 | 45 | 0.929 | 2.2 | Q2 | 55 | 3 | 1225 | 3 | 1998 |
| NEUROPEDIATRICS | 21 | 38 | 0.7 | 1.1 | Q3 | 46 | 5 | 536 | 13 | 1996 |
| EPILEPSIA | 19 | 21 | 0.633 | 6.6 | Q1 | 21 | 14 | 820 | 7 | 1996 |
| RESEARCH IN DEVELOPMENTAL DISABILITIES | 19 | 29 | 0.5 | 2.9 | Q1 | 37 | 8 | 401 | 20 | 1988 |
| JOURNAL OF CHILD NEUROLOGY | 18 | 29 | 0.643 | 2 | Q2 | 49 | 4 | 695 | 11 | 1998 |
| NEUROLOGY | 18 | 31 | 0.409 | 7.7 | Q1 | 31 | 9 | 1599 | 2 | 1982 |
| PEDIATRIC NEUROLOGY | 18 | 31 | 0.621 | 3.2 | Q1 | 42 | 6 | 751 | 9 | 1997 |
| PEDIATRICS | 18 | 21 | 0.667 | 6.2 | Q1 | 21 | 15 | 918 | 5 | 1999 |
| BRAIN & DEVELOPMENT | 16 | 24 | 0.552 | 1.4 | Q3 | 57 | 2 | 493 | 15 | 1997 |
| JOURNAL OF PEDIATRIC ORTHOPAEDICS | 16 | 28 | 0.571 | 1.4 | Q3 | 28 | 11 | 730 | 10 | 1998 |
| DISABILITY AND REHABILITATION | 15 | 23 | 0.652 | 2.1 | Q1 | 26 | 12 | 389 | 21 | 2003 |
| EUROPEAN JOURNAL OF PAEDIATRIC NEUROLOGY | 15 | 24 | 0.652 | 2.3 | Q2 | 41 | 7 | 381 | 22 | 2003 |
| ARCHIVES OF PHYSICAL MEDICINE AND REHABILITATION | 13 | 14 | 0.464 | 3.6 | Q1 | 14 | 22 | 865 | 6 | 1998 |
| CHILDS NERVOUS SYSTEM | 13 | 25 | 0.433 | 1.3 | Q3 | 28 | 10 | 219 | 44 | 1996 |
| AMERICAN JOURNAL OF PHYSICAL MEDICINE & REHABILITATION | 12 | 15 | 0.545 | 2.2 | Q2 | 15 | 20 | 213 | 47 | 2004 |
| CLINICAL BIOMECHANICS | 12 | 17 | 0.462 | 1.4 | Q3 | 17 | 19 | 184 | 54 | 2000 |
| DEVELOPMENTAL NEUROREHABILITATION | 12 | 19 | 0.667 | 1.1 | Q3 | 19 | 16 | 168 | 56 | 2008 |
| NEUROREHABILITATION AND NEURAL REPAIR | 12 | 15 | 0.706 | 3.7 | Q1 | 15 | 21 | 360 | 23 | 2009 |
| BRAIN | 11 | 13 | 0.393 | 10.6 | Q1 | 13 | 24 | 985 | 4 | 1998 |

Note(s): H_index: The h-index of the journal, which measures both the productivity and citation impact of the publications. IF: Impact Factor, indicating the average number of citations to recent articles published in the journal. JCR_Quartile: The quartile ranking of the journal in the Journal Citation Reports, indicating the journal's ranking relative to others in the same field (Q1: top 25%, Q2: 25%-50%, Q3: 50%-75%, Q4: bottom 25%). TP: Total Publications. TP_rank: Rank of Total Publications. TC: Total Citations. TC_rank: Rank of Total Citations. Average Citations: The average number of citations per publication. PY_start: Publication Year Start, indicating the year the journal started publication.

**Table S4 Publication and Citation Profiles of High-Impact Authors**

| **Author** | **h_index** | **g-index** | **m-index** | **PY_start** | **TP** | **TP_Frac** | **TP_rank** | **TC** | **TC_rank** |
| --- | --- | --- | --- | --- | --- | --- | --- | --- | --- |
| BOYD RN | 23 | 32 | 0.852 | 1999 | 32 | 6.59 | 2 | 1744 | 2 |
| GORDON AM | 21 | 34 | 0.778 | 1999 | 34 | 7.34 | 1 | 1223 | 6 |
| CIONI G | 19 | 23 | 0.576 | 1993 | 23 | 2.84 | 4 | 1490 | 3 |
| GUZZETTA A | 18 | 22 | 0.72 | 2001 | 22 | 2.48 | 6 | 1311 | 4 |
| ZIVIANI J | 16 | 23 | 0.889 | 2008 | 23 | 4.72 | 4 | 882 | 8 |
| GRAHAM HK | 14 | 16 | 0.467 | 1996 | 16 | 3.51 | 9 | 1230 | 5 |
| SAKZEWSKI L | 14 | 17 | 0.875 | 2010 | 17 | 3.34 | 8 | 753 | 10 |
| DESLOOVERE K | 13 | 16 | 0.481 | 1999 | 16 | 2.47 | 9 | 606 | 14 |
| ELIASSON AC | 13 | 16 | 0.481 | 1999 | 16 | 4.02 | 9 | 2277 | 1 |
| MERCURI E | 12 | 12 | 0.4 | 1996 | 12 | 1.42 | 14 | 876 | 9 |
| MIKATI MA | 12 | 30 | 0.414 | 1997 | 31 | 3.35 | 3 | 913 | 7 |
| BLEYENHEUFT Y | 11 | 14 | 0.579 | 2007 | 14 | 3.19 | 12 | 415 | 17 |
| DE VRIES LS | 11 | 11 | 0.393 | 1998 | 11 | 1.77 | 18 | 622 | 13 |
| GROENENDAAL F | 11 | 11 | 0.379 | 1997 | 11 | 1.77 | 18 | 719 | 12 |
| MOLENAERS G | 11 | 12 | 0.407 | 1999 | 12 | 1.83 | 14 | 588 | 15 |
| DUYSENS J | 10 | 12 | 0.455 | 2004 | 12 | 3.02 | 14 | 343 | 19 |
| FIORI S | 10 | 12 | 0.625 | 2010 | 12 | 1.43 | 14 | 733 | 11 |
| MAJNEMER A | 10 | 11 | 0.435 | 2003 | 11 | 2.17 | 18 | 398 | 18 |
| PRANGE L | 10 | 15 | 1.111 | 2017 | 20 | 1.86 | 7 | 261 | 20 |
| SASAKI M | 10 | 13 | 0.357 | 1998 | 13 | 1.88 | 13 | 419 | 16 |

Note(s): H_index: The h-index of the author, which measures both the productivity and citation impact of the publications. g_index: The g-index of the author, which gives more weight to highly-cited articles. m_index: The m-index of the author, which is the h-index divided by the number of years since the first published paper. TP: Total Publications. TP_rank: Rank of Total Publications. TC: Total Citations. TC_rank: Rank of Total Citations. Average Citations: The average number of citations per publication. PY_start: Publication Year Start, indicating the year the journal started publication.

**Table S5. The top 20 most cited papers.**

| **Paper** | **DOI** | **Total Citations** | **TC per Year** | **Normalized TC** |
| --- | --- | --- | --- | --- |
| ELIASSON AC, 2006, DEV MED CHILD NEUROL | 10.1017/S0012162206001162 | 1610 | 80.50 | 21.36 |
| SCHWARTZ MH, 2008, GAIT POSTURE | 10.1016/j.gaitpost.2008.05.001 | 523 | 29.06 | 11.61 |
| SPECCHIO N, 2022, EPILEPSIA | 10.1111/epi.17241 | 444 | 111.00 | 41.30 |
| BAX M, 2006, JAMA-J AM MED ASSOC | 10.1001/jama.296.13.1602 | 425 | 21.25 | 5.64 |
| GOULD DB, 2005, SCIENCE | 10.1126/science.1109418 | 413 | 19.67 | 6.40 |
| BOYD RN, 1999, EUR J NEUROL | 10.1111/j.1468-1331.1999.tb00031.x | 393 | 14.56 | 6.37 |
| WILEY ME, 1998, DEV MED CHILD NEUROL | 10.1111/j.1469-8749.1998.tb15369.x | 338 | 12.07 | 5.97 |
| WREN TAL, 2005, J PEDIATR ORTHOPED | 10.1097/00004694-200501000-00018 | 329 | 15.67 | 5.10 |
| RAJU TNK, 2007, PEDIATRICS | 10.1542/peds.2007-0336 | 326 | 17.16 | 5.99 |
| HEINZEN EL, 2012, NAT GENET | 10.1038/ng.2358 | 320 | 22.86 | 8.57 |
| HIMMELMANN K, 2005, ACTA PAEDIATR | 10.1111/j.1651-2227.2005.tb03071.x | 300 | 14.29 | 4.65 |
| EYRE JA, 2001, NEUROLOGY | 10.1212/WNL.57.9.1543 | 296 | 11.84 | 5.52 |
| DEVLIN AM, 2003, BRAIN | 10.1093/brain/awg052 | 288 | 12.52 | 5.29 |
| DAMIANO DL, 1998, ARCH PHYS MED REHAB | 10.1016/S0003-9993(98)90287-8 | 274 | 9.79 | 4.84 |
| HOON AH, 2009, DEV MED CHILD NEUROL | 10.1111/j.1469-8749.2009.03306.x | 248 | 14.59 | 7.83 |
| OEFFINGER D, 2008, DEV MED CHILD NEUROL | 10.1111/j.1469-8749.2008.03150.x | 240 | 13.33 | 5.33 |
| GANESAN V, 2000, DEV MED CHILD NEUROL | 10.1017/S0012162200000852 | 239 | 9.19 | 3.68 |
| ANCEL PY, 2006, PEDIATRICS | 10.1542/peds.2005-0091 | 236 | 11.80 | 3.13 |
| DELALANDE O, 2007, NEUROSURGERY | 10.1227/01.NEU.0000249246.48299.12 | 235 | 12.37 | 4.31 |
| GOODMAN R, 1994, J CHILD PSYCHOL PSYC | 10.1111/j.1469-7610.1994.tb01289.x | 235 | 7.34 | 2.73 |
